# Identification of Crohn's Disease-Related Biomarkers and Pan-Cancer Analysis Based on Machine Learning

**DOI:** 10.1155/mi/6631637

**Published:** 2025-04-04

**Authors:** Tangyu Yuan, Jiayin Xing, Pengtao Liu

**Affiliations:** ^1^School of Life Science and Technology, Shandong Second Medical University, Weifang, Shandong, China; ^2^School of Basic Medical Science, Shandong Second Medical University, Weifang, Shandong, China

**Keywords:** Crohn's disease, machine learning, pan-cancer, S100P

## Abstract

**Background**: In recent years, the incidence of Crohn's disease (CD) has shown a significant global increase, with numerous studies demonstrating its correlation with various cancers. This study aims to identify novel biomarkers for diagnosing CD and explore their potential applications in pan-cancer analysis.

**Methods**: Gene expression profiles were retrieved from the Gene Expression Omnibus (GEO) database, and differentially expressed genes (DEGs) were identified using the “limma” R package. Key biomarkers were selected through an integrative machine learning pipeline combining LASSO regression, neural network modeling, and Support Vector Machine-Recursive Feature Elimination (SVM-RFE). Six hub genes were identified and further validated using the independent dataset GSE169568. To assess the broader relevance of these biomarkers, a standardized pan-cancer dataset from the UCSC database was analyzed to evaluate their associations with 33 cancer types.

**Results**: Among the identified biomarkers, S100 calcium binding protein P (S100P) and S100 calcium binding protein A8 (S100A8) emerged as key candidates for CD diagnosis, with strong validation in the independent dataset. Notably, S100P displayed significant associations with immune cell infiltration and patient survival outcomes in both liver and lung cancers. These findings suggest that chronic inflammation and immune imbalances in CD may not only contribute to disease progression but also elevate cancer risk. As an inflammation-associated biomarker, S100P holds particular promise for both CD diagnosis and potential cancer risk stratification, especially in liver and lung cancers.

**Conclusion**: Our study highlights S100P and S100A8 as potential diagnostic biomarkers for CD. Moreover, the pan-cancer analysis underscores the broader clinical relevance of S100P, offering new insights into its role in immune modulation and cancer prognosis. These findings provide a valuable foundation for future research into the shared molecular pathways linking chronic inflammatory diseases and cancer development.

## 1. Introduction

Crohn's disease (CD) is a chronic, granulomatous inflammatory disorder that can affect any segment of the gastrointestinal tract, characterized by persistent and relapsing inflammation. Despite extensive research, the precise etiology of CD remains incompletely understood, and the disease typically follows a lifelong, progressive course. In addition to its impact on the digestive system, CD is increasingly recognized as a systemic disorder, often presenting with extraintestinal manifestations such as arthritis [1], bronchiectasis, Cryptogenic Organizing Pneumonia [2], erythema nodosum, and aphthous stomatitis [3]. Alarmingly, the global prevalence and incidence of CD have risen significantly, particularly among individuals aged 20–60 years [4–6], placing a substantial burden on healthcare systems worldwide.

Although the exact pathogenic mechanisms remain unclear, current evidence highlights that CD arises from a dysregulated immune response triggered by complex interactions between host genetic susceptibility, gut microbiota imbalance, and environmental factors [7]. This intricate pathogenic network underscores the urgent need to identify reliable biomarkers that can facilitate early diagnosis and improve disease monitoring.

Moreover, mounting evidence suggests that chronic intestinal inflammation in CD not only drives local tissue damage but may also increase the risk of various malignancies, including rectal cancer, small bowel cancer [8], cholangiocarcinoma [9], anal cancer [10], and urinary bladder cancer [11]. There are some studies that point out that there is no direct association between CD and certain cancers, but it may indirectly increase the risk of cancer through other pathways. For example, it has been found that CD is not directly related to gastric cancer, but the long-term inflammatory process may promote the development of gastric cancer [12]. In addition, medication for CD, especially overuse of medications, may increase the risk of blood cancers, skin cancers, and hepatosplenic T-cell lymphomas [13].

This inflammatory-carcinogenic link raises important questions about the shared molecular mechanisms underlying CD and tumorigenesis. To address these critical gaps, advanced bioinformatics and machine learning techniques were employed to systematically identify biomarkers associated with CD while also conducting pan-cancer analyses to assess their relevance across 33 cancer types. Through the integration of these analyses, the study seeks to uncover novel biomarkers with dual diagnostic potential for CD and predictive significance for cancer risk, offering new insights into the inflammatory cancer continuum.

## 2. Materials and Methods

### 2.1. Data Acquisition

**Figure 1:**
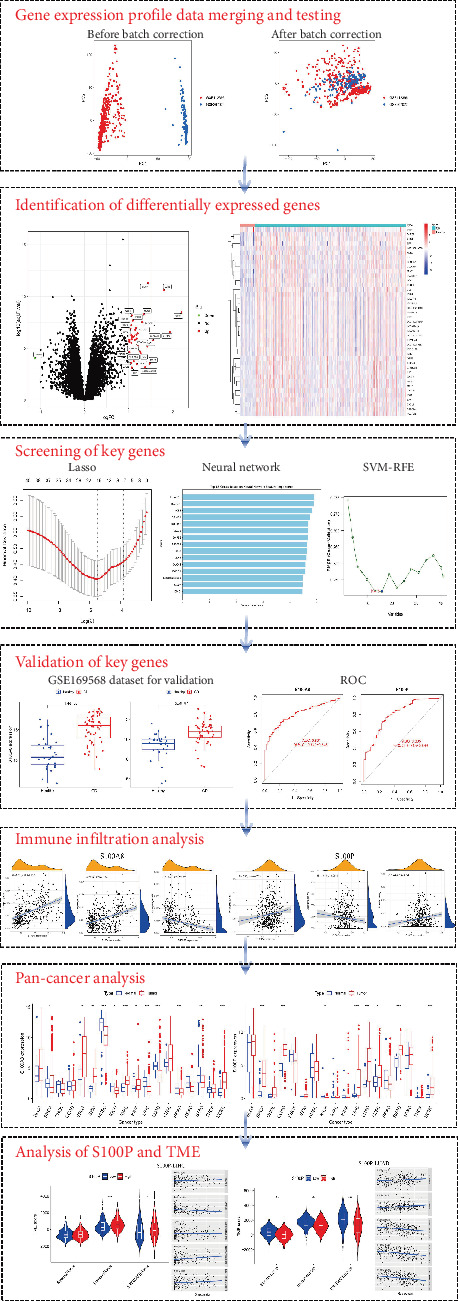
Overview of the analytical framework and corresponding results. AUC, area under the curve; ROC, receiver operating characteristic.

Relevant gene expression datasets were retrieved from the Gene Expression Omnibus (GEO, https://www.ncbi.nlm.nih.gov/geo/) and UCSC Xena (https://xenabrowser.net/) databases, as summarized in Table 1. The overall analysis workflow is illustrated in Figure 1.

### 2.2. Data Processing and Differential Expression Analysis

Datasets GSE112366 and GSE207022 were merged, with batch effects corrected using the “sva” package in R (v4.3.1). Differentially expressed genes (DEGs) between CD samples and healthy controls were identified using the “limma” package, with the cutoff criteria set at |log2 fold change (FC)| > 1 and *p*  < 0.05. The DEGs were visualized using heatmaps and volcano plots to depict global expression changes.

### 2.3. Enrichment Analysis

Functional enrichment analysis of DEGs was performed using the “clusterProfiler” package in R, covering Gene Ontology (GO), Kyoto Encyclopedia of Genes and Genomes (KEGG), and Disease Ontology (DO) annotations. GO analysis included three categories: molecular function (MF), cellular component (CC), and biological process (BPs). The KEGG pathway analysis provided insights into disease-related biological pathways, while DO analysis facilitated classification of DEGs within broader disease contexts, allowing comparative exploration of CD-associated gene signatures.

### 2.4. Hub Gene Selection

To identify potential hub genes, we applied an integrative machine learning framework combining LASSO regression, a neural network algorithm, and Support Vector Machine-Recursive Feature Elimination (SVM-RFE). LASSO regression (R package “glmnet”) applied L1 regularization to shrink irrelevant gene coefficients to zero [14, 15], retaining only the most informative DEGs. A neural network model, implemented in Python using “pandas”, “numpy”, “matplotlib.pyplot”, and “sklearn.neural_network”, automatically learned nonlinear patterns and complex gene interactions contributing to CD. SVM-RFE [16] (R packages “e1071”, “kernlab”, and “caret”) recursively eliminated weak predictors, ultimately selecting a refined panel of CD-relevant genes. The intersection of genes identified by all three methods was considered the final set of hub genes for further validation.

### 2.5. Hub Gene Validation

The independent dataset GSE169568 was used to validate the expression and diagnostic relevance of the identified hub genes. Gene expression differences between CD and healthy samples were visualized using box plots, while diagnostic performance was assessed using receiver operating characteristic (ROC) curves and area under the curve (AUC) metrics, with AUC > 0.80 considered indicative of strong diagnostic power.

### 2.6. Immune Infiltration Analysis

To estimate immune cell composition within CD samples, we applied the CIBERSORT algorithm in R, which uses linear support vector regression to deconvolute expression profiles into proportions of 22 immune cell types (LM22). This allowed exploration of the immune microenvironment and its relationship with identified hub genes.

### 2.7. Pan-Cancer Analysis

To investigate the broader relevance of hub genes across malignancies, gene expression profiles and clinical data spanning 33 tumor types were retrieved from the TCGA Pan-Cancer (PANCAN) dataset via the UCSC Xena platform. For each cancer type, differential gene expression between tumor and adjacent normal tissues was computed in R. Additionally, survival analysis using the “survival” package assessed associations between hub gene expression and patient prognosis across cancers. Correlations between hub gene expression and clinical characteristics were further analyzed in selected cancers.

### 2.8. Tumor Microenvironment Analysis

The tumor microenvironment (TME), particularly the relative abundance of immune and stromal components, plays a crucial role in cancer progression and prognosis. The ESTIMATE algorithm was used to compute immune and stromal scores from gene expression profiles, providing quantitative assessment of nontumor cell infiltration. To further characterize the immune landscape, CIBERSORT deconvolution was repeated (*n* = 1000 permutations) to ensure robustness [17]. Differences in immune functions and immune checkpoint expression between high and low hub gene expression groups were also examined to infer potential immunomodulatory roles of the identified biomarkers.

### 2.9. Statistical Analysis

All statistical analyses were conducted using R (v4.3.1). Differential expression analysis, correlation assessments, and diagnostic performance evaluations were performed using established statistical methods. Gene expression differences between CD and healthy samples were analyzed using limma's linear modeling and empirical Bayes methods. ROC curves were employed to evaluate diagnostic performance, with AUC > 0.80 considered indicative of strong predictive ability. Group comparisons were conducted using the Wilcoxon rank-sum test, with *p*  < 0.05 considered statistically significant.

## 3. Results

### 3.1. Identification of DEGs

**Figure 2:**
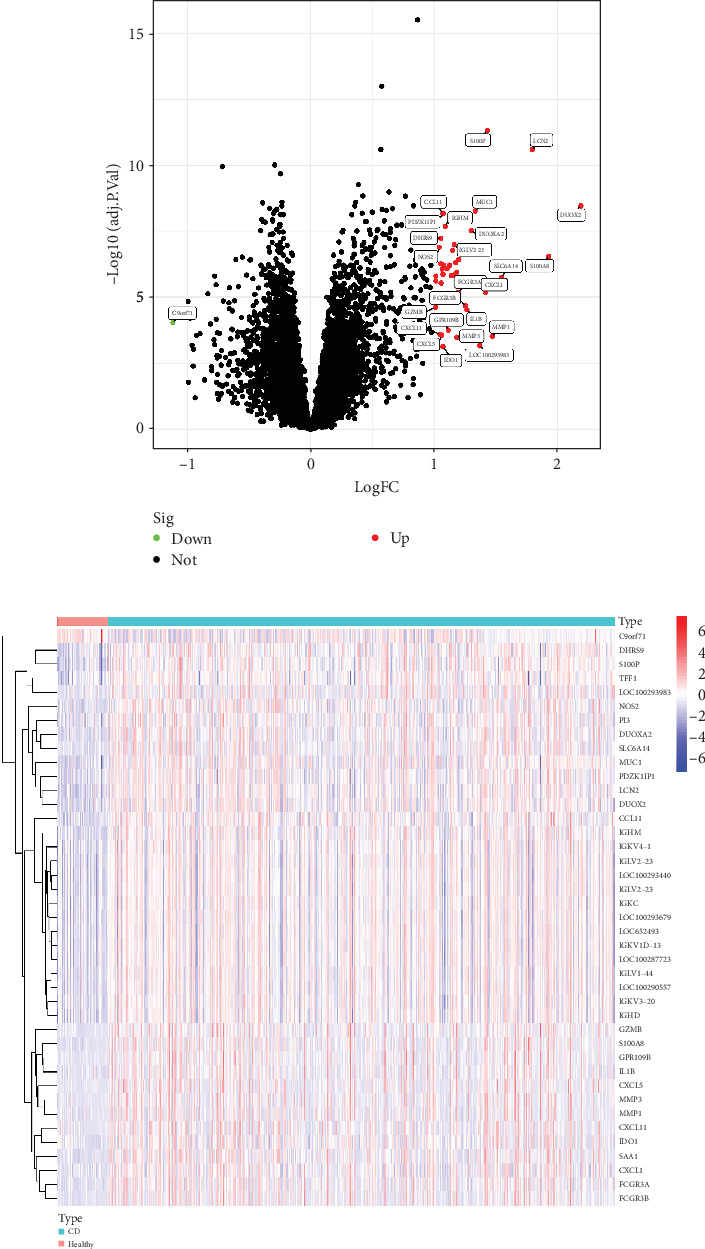
Identification of DEGs between CD patients and healthy controls (HCs). (A) Volcano plot depicting DEGs between CD patients and healthy controls. (B) Heatmap showing expression levels of DEGs in the merged dataset. CD, Crohn's disease; DEG, differentially expressed gene.

By integrating datasets GSE1112366 and GSE207022 and performing differential gene expression analysis, we identified a total of 41 DEGs (Figure 2A). These results indicate that a substantial number of genes exhibit increased expression levels in Crohn's disease (CD) patients compared to healthy controls. Figure 2B further illustrates the expression patterns of these DEGs across the merged dataset.

### 3.2. Enrichment Analysis of DEGs

To investigate the biological functions and pathway involvement of the identified DEGs, we conducted GO, KEGG, and DO enrichment analyses.

**Figure 3:**
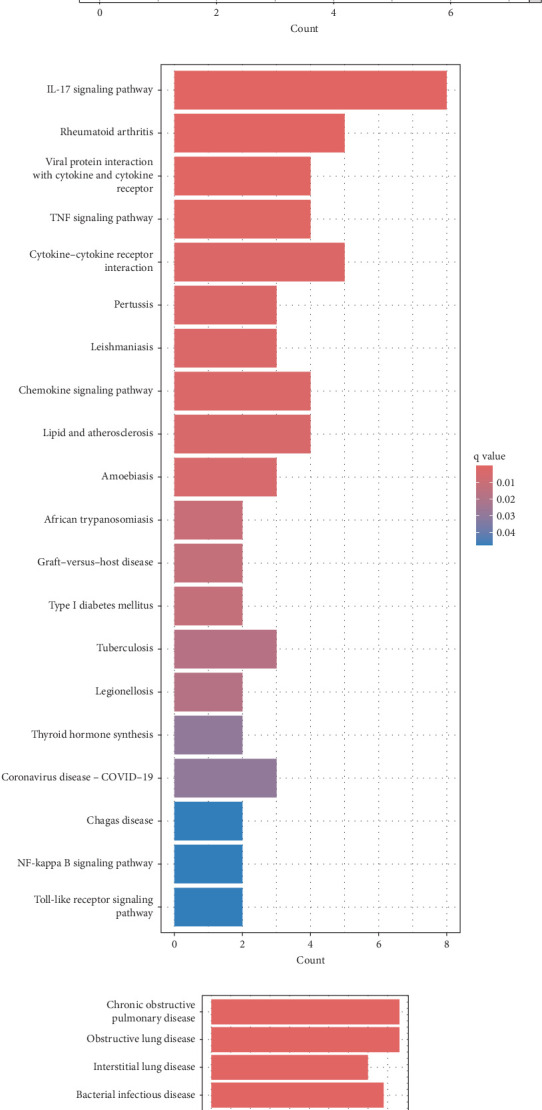
Functional enrichment analysis of DEGs. (A) GO enrichment analysis results divided into biological process (BP), cellular component (CC), and molecular function (MF). (B) KEGG pathway enrichment analysis results. (C) DO enrichment results. Adjusted *p*-value  < 0.05 was considered statistically significant. DEG, differentially expressed gene; DO, disease ontology; GO, gene ontology; KEGG, Kyoto Encyclopedia of Genes and Genome.

Gene Ontology enrichment analysis revealed that the DEGs are primarily enriched in BPs such as response to lipopolysaccharides, response to bacterial molecules, neutrophil chemotaxis, granulocyte migration, and leukocyte chemotaxis. These processes are closely linked to immune and inflammatory responses (Figure 3A).

Kyoto Encyclopedia of Genes and Genomes pathway analysis further identified five significantly enriched pathways, including the IL-17 signaling pathway, cytokine–cytokine receptor interaction, viral protein–cytokine interaction, TNF signaling pathway, and chemokine signaling pathway (Figure 3B). These pathways emphasize the key role of cytokines and chemokines in CD pathogenesis.

In addition, DO enrichment analysis highlighted that these DEGs are predominantly associated with diseases such as chronic obstructive pulmonary disease, bacterial infections, interstitial lung disease, and enteric diseases (Figure 3C).

### 3.3. Identification of Hub Genes

**Figure 4:**
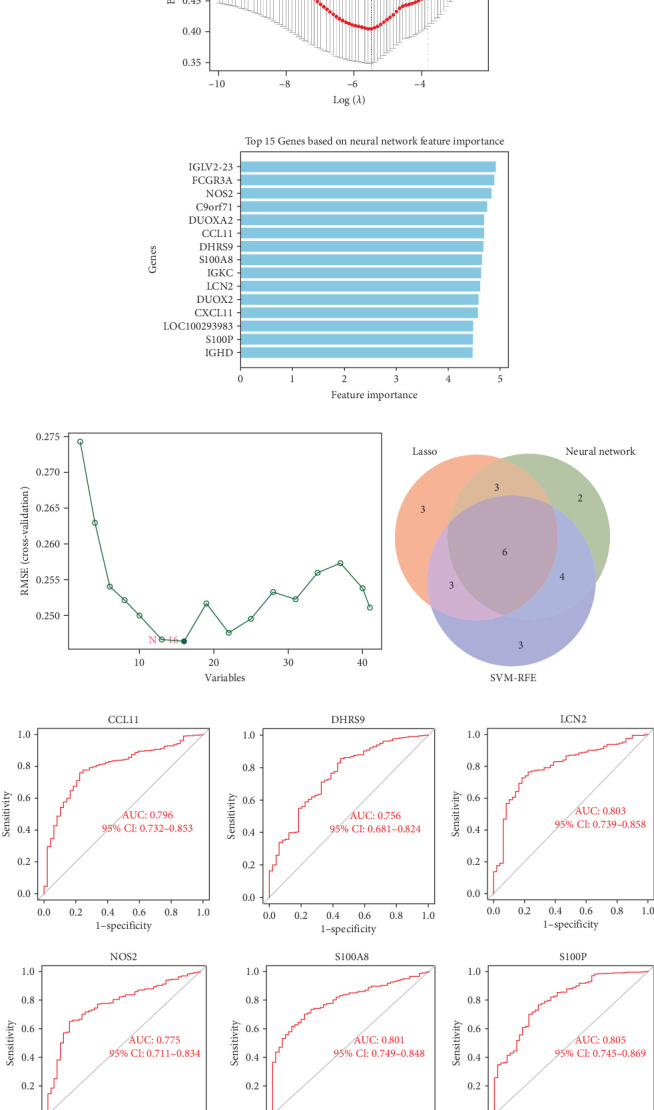
Identification and evaluation of key genes. (A) Genes selected by Lasso regression. (B) Genes identified by the neural network model. (C) Genes identified by SVM-RFE. (D) Venn diagram showing overlapping genes from the three models. (E) ROC curves evaluating the diagnostic performance of candidate hub genes. ROC, receiver operating characteristic; SVM-RFE, support vector machine-recursive feature elimination.

We employed three feature selection algorithms to screen hub genes from the DEGs. Lasso logistic regression (R package “glmnet”) identified 15 genes using the minimum lambda criterion (Figure 4A). A neural network model trained on the dataset identified 15 genes associated with CD (Figure 4B). SVM-RFE analysis (R package “e1071”) identified 16 genes relevant to CD diagnosis (Figure 4C). The hub genes selected for each model are shown in Table 2.

By intersecting the results of these three approaches, six overlapping genes were identified: LCN2, S100P, S100A8, CCL11, DHRS9, and NOS2 (Figure 4D). The diagnostic performance of these six genes was further assessed using ROC curves (Figure 4E), which highlighted S100A8, S100P, and LCN2 as promising biomarkers for CD diagnosis.

### 3.4. Validation of Hub Genes

**Figure 5:**
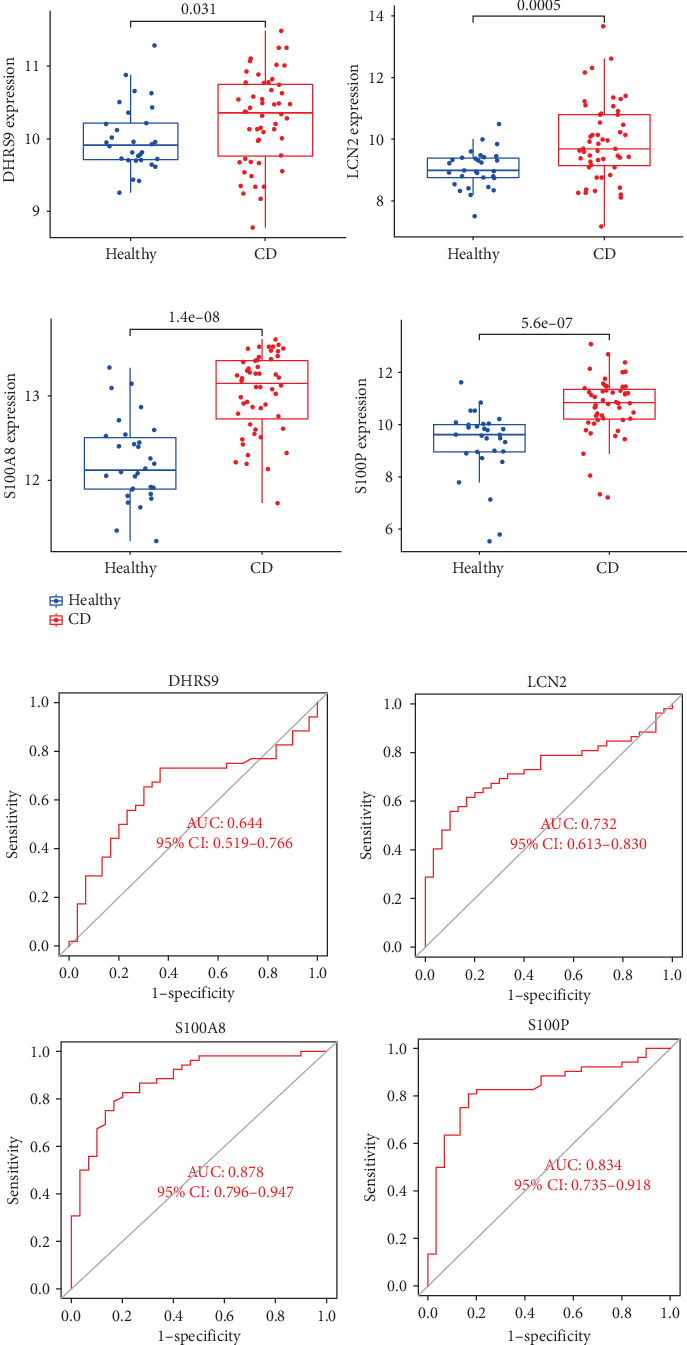
Validation of hub gene expression and diagnostic performance. (A) Boxplots comparing expression levels of hub genes in CD and HCs (GSE169568). (B) ROC curves evaluating diagnostic accuracy for the selected hub genes. *p* − value  < 0.05 was considered statistically significant. CD, Crohn's disease; HC, healthy control; ROC, receiver operating characteristic.

To validate the expression patterns and diagnostic utility of the hub genes, we analyzed the independent validation dataset GSE169568. Expression levels of S100P, S100A8, LCN2, and DHRS9 were significantly different between CD patients and healthy controls (Figure 5A). ROC analysis confirmed that S100P (AUC = 0.834) and S100A8 (AUC = 0.878) exhibited high diagnostic accuracy, while DHRS9 (AUC = 0.644) and LCN2 (AUC = 0.732) showed relatively lower diagnostic value (Figure 5B).

### 3.5. Immune Infiltration Analysis

**Figure 6:**
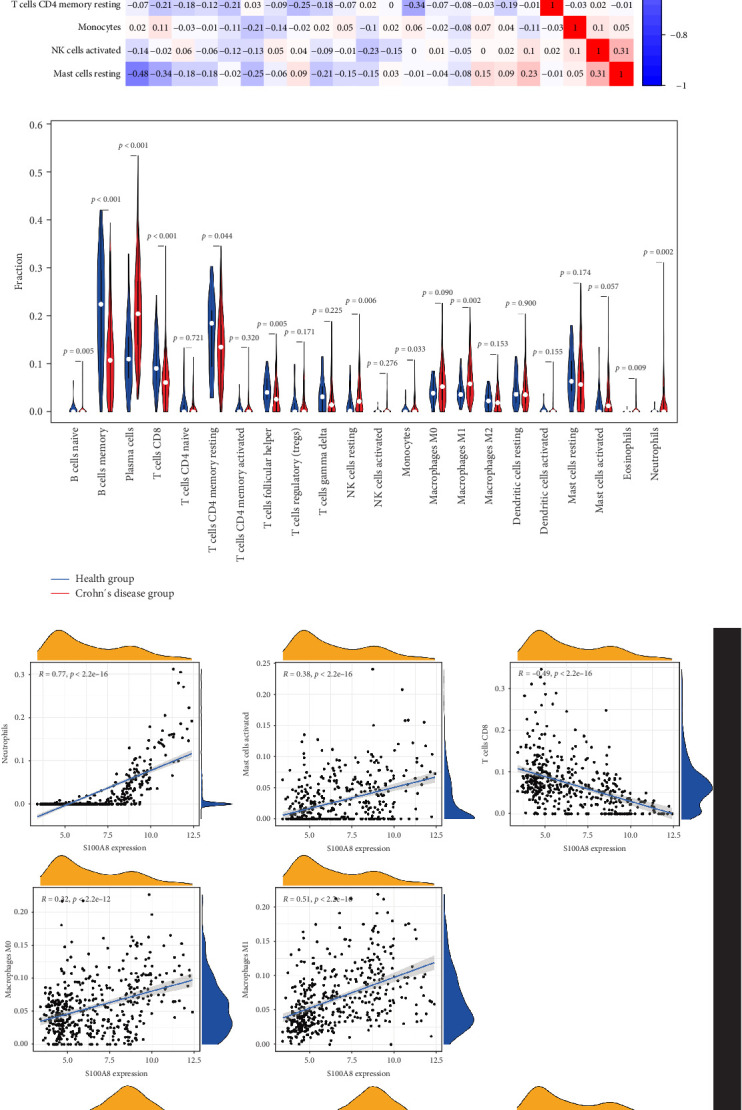
Immune infiltration analysis using CIBERSORT. (A) Immune cell composition across samples. (B) Differential expression of immune cells between CD and HCs. (C) Correlation between S100P, S100A8, and specific immune cells. CD, Crohn's disease; HC, healthy control.

We applied the CIBERSORT algorithm to estimate immune cell composition in CD and healthy samples (Figure 6A). Significant differences were observed between the groups (Figure 6B). In healthy controls, naive B cells, memory B cells, plasma cells, and follicular helper T cells exhibited higher abundance, indicating a role in immune surveillance and antibody production. In CD patients, there was a higher proportion of resting NK cells, M1 macrophages, eosinophils, and neutrophils, reflecting active inflammation and immune dysregulation.

Correlation analysis between hub gene expression and immune cell abundance revealed that S100P and S100A8 were positively correlated with neutrophil activation and mast cell activation, but negatively correlated with CD8+ T cells (Figure 6C). Additionally, S100A8 showed strong positive correlations with M0 and M1 macrophages, while S100P correlated positively with plasma cells but negatively with memory B cells.

### 3.6. Pan-Cancer Analysis

**Figure 7:**
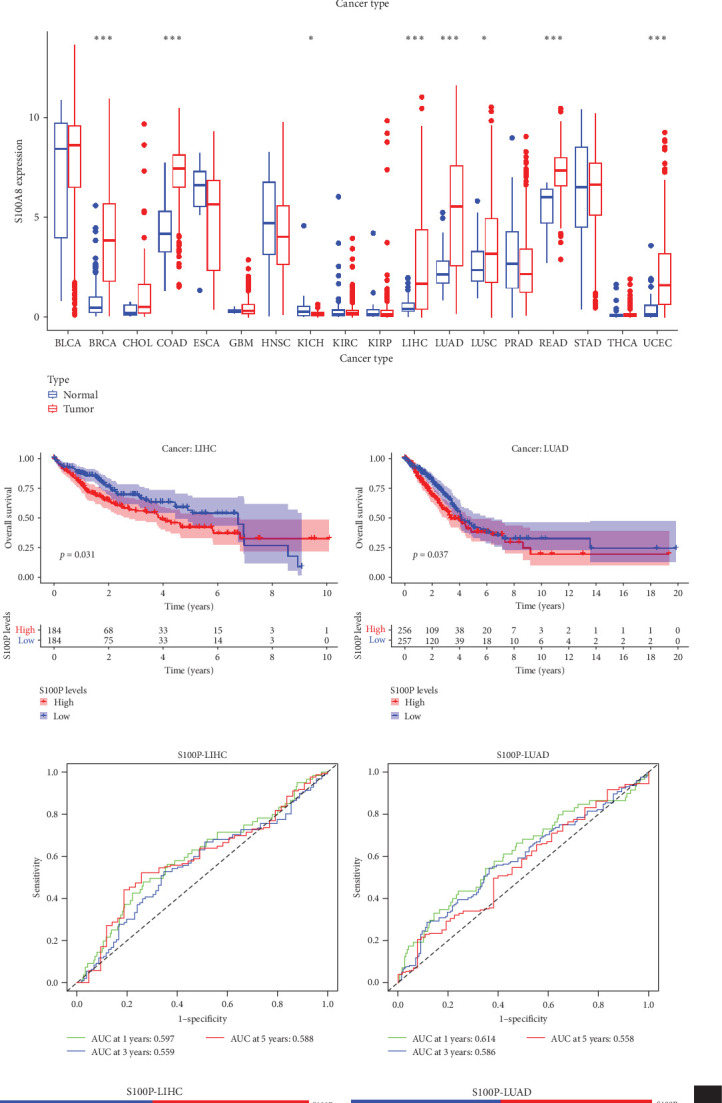
Pan-cancer analysis of S100P and S100A8. (A) Expression differences across tumor types. (B) Survival analysis of S100P in LIHC and LUAD. (C) ROC curves predicting survival based on S100P expression. (D) Association between S100P expression and clinical characteristics. *⁣*^*∗*^*p*  < 0.05,  ^*∗*^^*∗*^*p*  < 0.01, and  ^*∗*^^*∗*^*p*  < 0.001. LIHC, liver hepatocellular carcinoma; LUAD, lung adenocarcinoma; ROC, receiver operating characteristic.

In pan-cancer analysis, S100P and S100A8 exhibited significantly altered expression in 8 and 12 tumor types, respectively (Figure 7A).

Survival analysis demonstrated that S100P expression significantly influenced patient survival in liver hepatocellular carcinoma (LIHC) and lung adenocarcinoma (LUAD) (Figure 7B). ROC analysis further confirmed the predictive power of S100P for 1-year, 3-year, and 5-year survival in these cancers (Figure 7C).

Analysis of clinical factors revealed that S100P expression was significantly associated with gender, tumor M stage, and race in LUAD and gender in LIHC (Figure 7D).

### 3.7. TME Analysis of S100P

**Figure 8:**
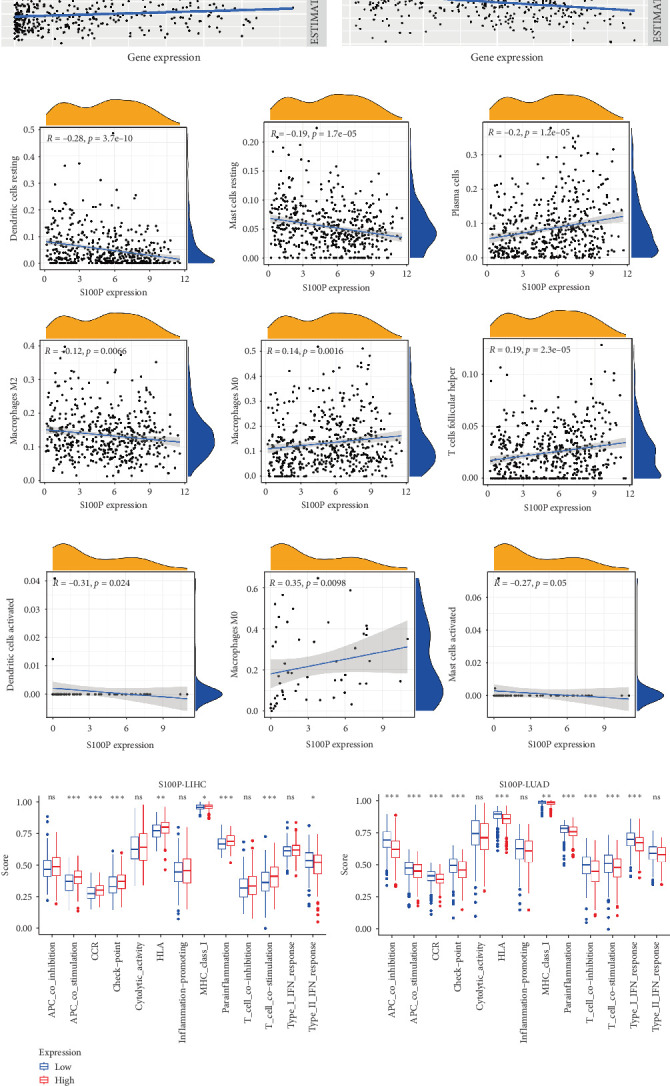
TME analysis of S100P in LIHC and LUAD. (A–F) Various immune components, functions, and checkpoints analyzed in relation to S100P expression. *⁣*^*∗*^*p*  < 0.05,  ^*∗*^^*∗*^*p*  < 0.01, and  ^*∗*^^*∗*^*p*  < 0.001. LIHC, liver hepatocellular carcinoma; LUAD, lung adenocarcinoma.

We further investigated the role of S100P in shaping the TME in LUAD and LIHC. In LUAD, high S100P expression correlated with lower immune scores, stromal scores, and estimate scores (Figure 8A) and showed a negative correlation with these scores but a positive correlation with DNAss and RNAss (Figure 8B). In LIHC, high S100P expression correlated with higher immune and estimate scores but no significant change in stromal score (Figure 8A).

S100P expression correlated negatively with resting dendritic cells, resting mast cells, and M2 macrophages but positively with M0 macrophages and T follicular helper cells (Figure 8C,D). Immune functions, chemokine receptor expression, and checkpoint expression differed significantly between S100P-high and S100P-low groups (Figure 8E,F).

## 4. Discussion

CD predominantly affects individuals under the age of 30, yet the incidence among elderly populations is steadily increasing [18]. Notably, Germany reports the highest incidence, reaching 322 cases per 100,000 individuals [19]. Despite advancements in therapeutic strategies, over 80% of CD patients experience postoperative recurrence, posing a considerable challenge for clinical management [20, 21]. As treatment options continue to evolve, the identification of reliable biomarkers to predict therapeutic response is crucial. In this study, we applied machine learning algorithms to identify and validate S100P and S100A8 as potential biomarkers for CD, thereby offering novel insights into its diagnosis and management.

Although previous studies have identified various biomarkers for CD [22, 23], our study applied advanced machine learning approaches to uncover novel candidates, ultimately highlighting S100P and S100A8. The utility of these genes was subsequently confirmed using an independent dataset, further supporting their robustness as potential biomarkers.

The pathogenesis of CD remains incompletely understood, but it is widely recognized as a disorder driven by immune dysfunction and microbial imbalances [7]. In our enrichment analysis of DEGs, we observed significant enrichment in pathways related to responses to lipopolysaccharides and bacterial molecules, underscoring the critical role of microbial dysbiosis in CD development. Furthermore, we identified the involvement of the TNF signaling pathway and Toll-like receptor (TLR) signaling pathway, which has previously been linked to immune-related diseases such as idiopathic pulmonary fibrosis [24, 25].

Integrating enrichment analysis with immune infiltration profiling, we found that S100P and S100A8 overexpression in CD patients may lead to aberrant activation of the IL-17 signaling pathway, triggering excessive production of cytokines and chemokines. These inflammatory mediators promote neutrophil recruitment and prolong their lifespan [26–28], creating a self-sustaining inflammatory loop that exacerbates CD pathology. Therefore, targeting neutrophil activation may represent a promising therapeutic avenue for CD. This mechanistic link further strengthens the rationale for S100P and S100A8 as key biomarkers.

Previous studies have also suggested associations between CD and certain cancers, particularly colorectal cancer [29], anal cancer [10], oral cavity cancer, and breast cancer [30]. Expanding upon this, we conducted a pan-cancer analysis to examine the relevance of our identified biomarkers across 33 cancer types. This analysis revealed significantly altered expression of S100P in LIHC and LUAD, with strong correlations between S100P expression and patient survival outcomes. Moreover, S100P expression was associated with specific clinical features in both LIHC and LUAD. These findings are consistent with previous studies showing that patients with CD lead to an increased risk of developing lung cancer [31, 32]. Chronic inflammation, steroid use, and hepatic steatosis in CD promote cirrhosis, which may increase cancer risk [33]. Our results thus offer valuable insights into the potential interplay between CD and cancers beyond the gastrointestinal tract, particularly in the lung and liver.

S100P and S100A8 both belong to the S100 protein family, which regulates diverse biological processes, including cell differentiation, proliferation, migration, and apoptosis, largely through interactions with key signaling proteins such as p53, *β*-catenin, and NF-*κ*B [34, 35]. Numerous studies have linked dysregulated S100 protein expression to lung cancer initiation and progression [36–38]. High S100P expression in metastatic lung tumors is also associated with poorer survival [39–41]. Additionally, recent research highlights S100P as a ferroptosis inhibitor, promoting hepatocellular carcinoma by reprograming lipid metabolism [42].

In LIHC, our TME analysis revealed significant shifts in immune cell populations, though no substantial changes were observed in stromal components. High S100P expression suppressed dendritic cell and mast cell abundance while promoting M0 macrophage infiltration, contributing to an immunosuppressive environment conducive to tumor progression. In LUAD, low S100P expression correlated with elevated B cells, M0 macrophages, M1 macrophages, and dendritic cells, highlighting S100P's potential role in modulating macrophage polarization and thus shaping the TME. Additionally, S100P expression influenced immune checkpoint levels, suggesting its role in immune evasion. As antigen-presenting cells, dendritic cells are central to T cell priming; however, tumor cells often suppress dendritic cell function to escape immune surveillance [43]. Together, these findings underscore S100P's relevance as both a diagnostic and therapeutic target in LIHC and LUAD.

## 5. Limitations

This study has several limitations. First, the validation dataset used was relatively small compared to the training datasets, which may reduce the generalizability of our findings. Second, our pan-cancer analysis focused only on cancer types where hub genes exhibited statistically significant correlations with overall survival (*p* < 0.05). Consequently, we may have overlooked potential associations between S100P/S100A8 and other cancers, particularly those with smaller sample sizes. Existing literature suggests that CD is associated with increased risks of colorectal cancer, skin cancer, hematologic malignancies, and urinary tract cancer, which were not comprehensively explored in this study.

## 6. Conclusion

In summary, S100P and S100A8 emerged as promising biomarkers for CD, providing novel directions for its diagnosis and potential treatment stratification. Additionally, S100P demonstrated potential as a prognostic and therapeutic biomarker in liver and lung cancers, further expanding its clinical relevance. These findings not only enhance our understanding of the molecular mechanisms underlying CD but also offer a bridge to explore its relationship with oncogenesis, especially in LIHC and LUAD.

## Figures and Tables

**Table 1:** Summary of datasets used in this study.

Database	Dataset	Usage	Healthy samples	Disease samples	Other samples	Total samples
GEO	GSE112366	CD experimental group	26	362	—	388
	GSE207022	CD experimental group	23	125	—	148
	GSE169568	CD validation group	30	52	123	205

UCSC	A standardized pan cancer dataset of 33 types of cancer					
	TCGA pan cancer (PANCAN, *N* = 12,591) dataset					

Abbreviation: GEO, Gene Expression Omnibus.

**Table 2:** Gene names selected by three models.

Model	Gene		Model	Gene		Model	Gene	
Lasso	S100P	CXCL1	NN	NOS2	DHRS9	SVM-RFE	S100P	PDZK1IP1
	LCN2	GZMB		IGHD	C9orf71		IGHM	S100A8
	CCL11	C9orf71		S100P	FCGR3A		NOS2	DUOX2
	IGHM	GPR109B		CXCL11	DUOXA2		LCN2	SLC6A14
	DHRS9	MMP1		DUOX2	CCL11		PI3	DUOXA2
	NOS2	IGLV2-23		LCN2	IGLV2-23		CCL11	DHRS9
	S100A8	LOC100293983		IGKC	LOC100293983		TFF1	CXCL11
	TFF1	—		S100A8	—		CXCL1	IGHD

Abbreviation: SVM-RFE, support vector machine-recursive feature elimination.

## Data Availability

All code is saved on the GitHub website (https://github.com/YuanTangyu/Crohn-s-disease.git). The datasets used in this study are available in the Gene Expression Omnibus (GEO) database (https://www.ncbi.nlm.nih.gov/geo/) under the accession numbers “GSE112366”, “GSE207022,” and “GSE169568”. The standardized pan cancer dataset of 33 types of cancer used in this study can be obtained from the University of California Santa Cruz (UCSC) database (https://xenabrowser.net/), which includes clinical information and gene expression data of the samples.

## Abbreviations

ACC: Adrenocortical carcinoma
AUC: area under curve
BLCA: bladder urothelial carcinoma
BRCA: breast cancer
CD: Crohn's disease
CESC: cervical squamous cell carcinoma and endocervical adenocarcinoma
CHOL: Cholangiocarcinoma
COAD: Colorectal adenocarcinoma
DEGs: Differentially expressed genes
DLBC: Diffuse large B-cell lymphoma
DO: Disease Ontology
ESCA: esophageal carcinoma
GBM: glioblastoma multiforme
GEO: gene expression omnibus
GO: gene ontology
HCs: healthy controls
HNSC: head and neck squamous cell carcinoma
KEGG: Kyoto Encyclopedia of Genes and Genomes
KICH: kidney chromophobe
KIRC: kidney renal clear cell carcinoma
KIRP: kidney renal papillary cell carcinoma
LAML: acute myeloid leukemia
LGG: low grade glioma
LIHC: liver hepatocellular carcinoma
LUAD: lung adenocarcinoma
LUSC: lung squamous cell carcinoma
MESO: malignant mesothelioma
OV: ovarian cancer
PAAD: pancreatic adenocarcinoma
PCPG: pheochromocytoma and paraganglioma
PRAD: prostate adenocarcinoma
READ: rectal adenocarcinoma
ROC: receiver operating characteristic
S100A8: S100 calcium binding protein A8
S100P: S100 calcium binding protein P
SARC: Sarcoma
SKCM: cutaneous melanoma
STAD: stomach adenocarcinoma
SVM-RFE: support vector machine-recursive feature elimination
TGCT: testicular germ cell tumor
THCA: thyroid cancer
THYM: thymoma
TME: tumor microenvironment
UCEC: uterine corpus endometrial carcinoma
USC: uterine carcinosarcoma
UVM: uveal melanoma
